# The Link Between Metabolic Dysfunction-Associated Steatotic Liver Disease and Gastroesophageal Reflux Disease

**DOI:** 10.7759/cureus.71095

**Published:** 2024-10-08

**Authors:** Priyata Dutta, Anika Annoor, Proma Dey, Jakia Sultana, Mobin Ibne Mokbul, Sadia Afrin Naurin, Ritwik Roy, Sultana Y Simona, Jui Dutta, Tanusree Mazumder, Farjana Masud

**Affiliations:** 1 Internal Medicine, Trinity Health, Ann Arbor, USA; 2 Oncology, Texas Oncology, Dallas, USA; 3 Internal Medicine, Chittagong Medical College, Chattagram, BGD; 4 Internal Medicine, Comilla Medical College, Cumilla, BGD; 5 Internal Medicine, Dhaka Medical College, Dhaka, BGD; 6 Internal Medicine, Ibrahim Medical College, Dhaka, BGD; 7 Internal Medicine, Ragib-Rabeya Medical College and Hospital, Chittagong, BGD; 8 Internal Medicine, Khulna Medical College, Khulna, BGD; 9 Family Medicine, Zainul Haque Sikder Women's Medical College and Hospital, Dhaka, BGD; 10 Internal Medicine, Shaheed Ziaur Rahman Medical College, Dhaka, BGD

**Keywords:** bmi, gerd, masld, obesity, risk factor

## Abstract

Metabolic dysfunction-associated steatotic liver disease (MASLD), formerly nonalcoholic fatty liver disease (NAFLD), and gastroesophageal reflux disease (GERD) are prevalent chronic conditions with escalating global incidence. This study delves into the intricate interplay between MASLD and GERD. The primary objective is to comprehensively explore the association between MASLD and GERD, investigating how various factors contribute to the coexistence and potential exacerbation of these conditions. We conducted a literature search in PubMed and Google Scholar of only human studies over the past 10 years. The search included systematic review, meta-analysis, editorial, and cross-sectional studies of patients with MASLD and GERD. The prevalence of GERD in patients with MASLD was higher, with various risk factors coming into play. Obesity was identified as an independent risk factor for both GERD and MASLD. However, obese patients predominantly had higher disease progression. Lifestyle factors like physical activity and dietary modifications emerge as promising strategies to mitigate risk.

## Introduction and background

Metabolic dysfunction-associated steatotic liver disease (MASLD), previously known as nonalcoholic fatty liver disease (NAFLD), is a chronic liver condition caused by the accumulation of fat in the liver, often linked to obesity and insulin resistance. It is the most predominant chronic liver disease globally, attributable to the obesity pandemic and excessive calories [1,2]. Non-alcoholic fatty liver disease is defined as a subtype of fatty liver characterized by the accumulation of fat in the liver, not attributed to excessive alcohol consumption or other factors causing steatosis [3]. The new term, 'metabolic-dysfunction associated steatotic liver disease,' abbreviated as MASLD, was introduced recently in an international expert review [4]. Beyond a crude distinction between non-steatohepatitis and steatohepatitis, this new nomenclature seeks to expand the methodology for disease assessment and severity stratification. The use of MASLD reflects a more comprehensive understanding of the complex interactions between metabolic factors and liver health in individuals with fatty liver disease [5].

This disease develops through several complex processes, including the body's inability to use insulin properly, harmful fat buildup, and inflammation in the liver. Hepatic stellate cell (HSC) activation causes fibrogenesis. Some are relatively well-established, and some are less elucidated [6]. Much like how clogged pipes can cause pressure to build up in a house's plumbing, excess fat in the liver can cause damage over time, leading to liver diseases like MASLD.

Gastroesophageal reflux disease (GERD) is one of the most common health problems in the Western world and is defined as a chronic condition with recurrent exposure to gastric acid in the esophagus due to reduced lower esophageal sphincter tone contributing to various symptoms [7]. It is a prevalent condition in the Middle East and Africa. However, there is a lack of data on its management, and more clinical trials need to be done [8]. Its incidence has rapidly risen, and its estimated prevalence varies from 10% to 30% in the general population [9]. There is a significant association between obesity and an increased risk of symptoms related to GERD, erosive esophagitis, and esophageal adenocarcinoma. As weight increases, the risk of these disorders also increases progressively. Research suggests that the incidence of obesity in Western populations has risen alongside the occurrence of esophageal adenocarcinoma, indicating that this might play a crucial role in the trend [10,11].

However, studies show the severity and prevalence of GERD symptoms in patients with NAFLD are high, which is associated with increased levels of serum triglyceride (TG) and serum cholesterol but not with simple obesity. Findings from studies on the Japanese population also point out that the risk of Barrett's esophagus is primarily influenced by abdominal obesity, particularly the visceral fat area, rather than by simple obesity [12,13].

Objective

Several mechanisms may explain the relationship between abdominal obesity and the risk of GERD/Barrett's esophagus and esophageal adenocarcinoma, which are discussed in the subsequent sections of the article [14]. The primary objective of this narrative review is to comprehensively summarize and explore the association between MASLD and GERD, investigating how various factors contribute to the coexistence and potential exacerbation of these conditions. Understanding the connection between these two prevalent conditions is crucial, as both have high rates of morbidity and affect millions worldwide, with potential implications for public health strategies and patient care.

Methodology

We conducted an extensive search on PubMed, Medline, Embase, and Google Scholar, and the search strategy included the use of the following keywords: 'metabolic dysfunction-associated steatotic liver disease,’ ‘non-alcoholic fatty liver disease,’ ‘obesity,’ and ‘gastroesophageal reflux disease.’ Our inclusion criteria for this review were human studies, clinical trials, cross-sectional and cohort studies, systematic reviews and meta-analyses, studies in the English language, and sufficient data on MASLD, GERD, and obesity. 

## Review

MASLD and its pathophysiology

The updated terminology, MASLD, highlights the importance of metabolic dysfunction, removing the previous requirement for the exclusion of significant alcohol intake or other chronic liver diseases in the diagnosis [15]. The pathogenesis of MASLD involves a paradigm shift from the traditional 'two-hit' hypothesis to the 'multiple parallel hits' theory. This new perspective encompasses factors such as insulin resistance (IR), lipotoxicity, genetic elements, and endoplasmic reticulum (ER) stress [2]. Accumulation of toxic lipid species has a significant effect on MASLD. The primary event is the buildup of free fatty acids (FFAs) inside the hepatic cell, sourced from three main origins. First, excessive mobilization of FFAs from adipose tissue, driven by IR, contributes about 60% of hepatic TGs [6]. Studies in human models have identified inhibited insulin-associated PI3K/Akt/mTOR pathways and an association between hyperinsulinemia, lower insulin sensitivity, and MASLD. Adipose tissue, functioning as an endocrine organ, releases extracellular vesicles (EVs) containing microRNAs (miRNAs) that impact IR in hepatocytes. Proinflammatory macrophages in obesity also contribute to EV secretion, further affecting insulin sensitivity [2]. Second, de novo lipogenesis (DNL) is responsible for approximately 26% of deposited TGs, with hyperinsulinemia upregulating DNL through sterol regulatory element binding protein-1c (SREBP-1c). Third, dietary lipids make up around 15% of liver TGs, with evidence suggesting saturated fats like palmitate and stearate are more harmful. The liver processes FFAs through two mechanisms: oxidation mainly in mitochondria and re-esterification in very-low-density lipoprotein (VLDL) export. Increased fatty acid (FA) uptake, DNL, decreased FFA oxidation, and VLDL export, break the TG homeostasis, which ultimately causes hepatic steatosis. When FFA disposal mechanisms are overburdened, the generated reactive oxygen species (ROS) and toxic lipid species create lipotoxicity [6].

Moreover, inflammasomes, or intracellular pattern recognition receptors (PRRs), trigger the buildup of cytokines like IL-1b and IL-18, which ultimately contribute to NAFLD pathogenesis. While NLRP3 inflammasome components are minimally expressed in healthy hepatocytes, their significant growth is observed in human metabolic dysfunction-associated steatohepatitis (MASH). Immune cells like Kupffer cells, monocytes, neutrophils, T-helper cells, and cytotoxic CD8+ T cells also play crucial roles. Activated Kupffer cells cause inflammation by secreting CCL2, IL-1b, tumor necrosis factor-alpha (TNF-α), and IL-6 and recruiting monocytes and neutrophils. The cells show imbalances in MASH, with an excess of Th1-derived interferon-gamma (IFN-γ) and Th17-derived IL-17 and an absence of Th2-derived IL-4, IL-5, and IL13. These result in decreased steatosis, IR, and inflammation and ultimately trigger HSC activation. The TAZ (a transcriptional regulator), hedgehog (HH) ligands, transforming growth factor-beta (TGF-β), bone morphogenetic protein 8B (BMP8B), and osteopontin also have crucial roles in the activation of HSCs. This activation entails the increased expression of several genes, such as smooth muscle actin (a-SMA), collagen-1a1, tissue inhibitor of metalloproteinases (TIMP-1 and 2), and TGF-β, which ultimately contribute to fibrogenesis and impair liver structure and function [6]. Genetic factors also influence MASLD, particularly genes related to lipid droplet biology such as patatin-like phospholipase domain-containing 3 (PNPLA3), transmembrane six superfamily member 2 (TM6SF2), 17b-hydroxysteroid dehydrogenase type 13 (HSD17B13), membrane-bound O-acyltransferase domain-containing 7 (MBOAT7), and glucokinase regulator (GCKR). The PNPLA3 I148M variant is resistant to degradation, leading to its accumulation on lipid droplets, hindering the release of TGs from these droplets, and contributing to hepatic fat buildup and the progression of MASLD [6].

**Figure 1:**
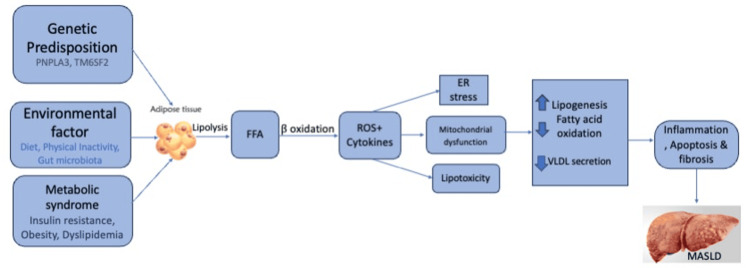
Demonstration of the pathophysiology of MASLD and the various factors predisposing to its progression MASLD: Metabolic dysfunction-associated steatotic liver disease, FFA: Free fatty acid, ROS: Reactive oxygen species, ER: Endoplasmic reticulum, VLDL: Very-low-density lipoprotein Illustration created by the authors.

Furthermore, imbalances in gut microbiota, disrupted bile acid homeostasis, gut alcohol buildup, and fructose processing in the intestines are implicated in altering susceptibility to MASLD. The presence of alcohol-producing bacteria in the microbiome, along with elevated blood-ethanol levels, suggests a contribution of these microbiota to MASLD. Recent research has identified a notable presence of a high-alcohol-producing strain of Klebsiella pneumoniae in many individuals with MASLD [6]. Figure 1 illustrates the pathophysiology of MASLD. 

Pathophysiology of GERD

Gastroesophageal reﬂux disease is a chronic condition caused by abnormal exposure of the distal esophagus to refluxed gastric and duodenal content [16]. This condition can cause manifestations like dysphagia, regurgitation, belching, and heartburn. Dyslipidemia, with its high blood lipid levels, can undermine the lower esophageal sphincter's (LES) function, heightening the risk of GERD by increasing the esophagus's exposure to gastric acid. The change in lipid levels could weaken the LES' ability to block the backflow of stomach contents. This suggests that an altered lipid profile weakens the LES' defense against reflux. Similarly, hyperglycemia, characterized by elevated blood sugar levels, may also interfere with GERD prevention by modulating the LES' transient relaxation. Moreover, hyperglycemia often leads to autonomic neuropathy, which can delay gastric emptying, thereby increasing the likelihood of GERD as stomach contents remain longer, raising the chance of reflux [17].

Research has shown that certain lifestyle factors, such as smoking and dietary habits, specifically, the frequent consumption of spirits, sweets, or white bread, are linked to an increased risk of reflux symptoms [7]. Smoking can impair the function of the LES by relaxing its smooth muscle, making it easier for stomach acid to backflow in the esophagus and trigger GERD symptoms. Furthermore, smoking diminishes saliva production, which is vital for neutralizing and washing away esophageal acid due to its bicarbonate content. On the other hand, indulging in spirits, sweets, or white bread might heighten stomach acidity or slow down stomach emptying, thereby aggravating GERD symptoms [18]. Conversely, physical activity and fruit consumption have been observed to offer a protective effect against these symptoms. As such, adopting lifestyle changes, such as managing weight and modifying diet, could significantly mitigate the risk of experiencing GERD symptoms [18,19].

The link between GERD, MASLD, and obesity

Obesity, a chronic multifactorial disease, is currently a global epidemic, posing a high burden on human health and the economy. In obesity, fatty tissue undergoes expansion by hypertrophy and hyperplasia, and inadequate vascularization leads to hypoxia and adipocyte apoptosis/necrosis, resulting in adipocyte dysfunction [20,21]. These lead to altered adipokines, which are related to systemic inflammation, irregular FA influx, lipolysis, and IR [1].

In obesity, a sustained disproportionality persists between the intake and expenditure of calories. The adipose tissue’s capacity for fat storage is optimized, and this causes abnormal fat deposition in different vital organs such as the liver, heart, and kidneys. These outcomes lead to the onset of persistent metabolic conditions such as NAFLD, nonalcoholic steatohepatitis (NASH), type 2 diabetes, cardiovascular diseases, and different cancers [22].

Obesity frequently impacts the liver, the primary site for processing glucose and lipids. When the capacity of the liver to synthesize and take up fatty acids surpasses its capacity to oxidize and export them, surplus lipids accumulate in the liver tissue, leading to MASLD. Liver injury related to NAFLD can eventually lead to NASH, hepatic failure, and cirrhosis. Hepatoma and cardiovascular diseases pose substantial risks in individuals with MASLD [22,23]. Due to obesity, many individuals develop obstructive sleep apnea and hypoxia during sleep time, which exacerbates the advancement of MASLD [24].

Research on bariatric surgery demonstrates a distinct, BMI-dependent relationship with MASLD risk, indicating that 85% to 95% of individuals with severe obesity develop MASLD. That's why individuals with obesity/overweight and metabolic diseases are recommended to do routine screening for MASLD. Furthermore, screening for cardiovascular diseases is also recommended in patients with MASLD [22,25].

The hallmark of metabolic syndrome, central obesity, is associated with both GERD and MASLD. Obesity may put the stomach under direct mechanical pressure, raising intragastric pressure and increasing the likelihood that the LES may relax, which will result in reflux. There has been recent research indicating a relationship between waist circumference (WC) and body mass index (BMI), intragastric pressure, and the gastroesophageal pressure gradient. It has been demonstrated that the WC has a stronger correlation than BMI [26]. Thus, obesity around the abdomen may have a significant role in the development of GERD and be a significant risk factor for Barrett’s esophagus. The biochemical activity of adipose tissues, especially visceral fatty tissue, which releases adipokines like TNF-α and IL-6, may be linked to an additional mechanism. These adipokines may play a role in the development of Barrett’s esophagus or subsequent malignancy. The IL-6 decreases the contraction of the esophagus and hinders the clearance of acid. All this contributes to the pathogenesis of GERD. The antioxidant property in NAFLD patients is impaired, thus causing oxidative injury in the esophageal mucosa. Finally, there is more fat in NAFLD patients, as they are usually obese [27,9].

**Figure 2:**
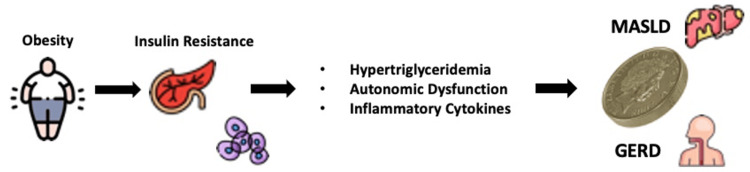
How obesity contributes to both MASLD and GERD MASLD: Metabolic dysfunction-associated steatotic liver disease, GERD: Gastroesophageal reflux disease Illustration created by the authors.

Adipose tissues release leptin primarily, and body fat content is directly correlated with elevated serum levels of this adipokine. It has been demonstrated that in Barrett-derived esophageal adenocarcinoma cells, leptin promotes cell proliferation and inhibits apoptosis. The characteristics of increased proliferation and decreased apoptosis, which are commonly seen in Barrett’s esophagus, are critical for the emergence of malignancy because they promote the accumulation and upkeep of genetic defects. Men whose blood leptin levels are in the top quartile have a threefold greater risk of Barrett’s esophagus, according to Kendall et al. [26]. As hypertriglyceridemia is a part of the metabolic syndrome, NAFLD patients frequently have it. Surprisingly, studies suggest that TGs could affect the LES' tone and could be the common cause of both NAFLD and GERD. Furthermore, a malfunctioning autonomic nervous system may be the connection between GERD and NAFLD. Research has revealed that autonomic disruption was more common in NAFLD patients. Additionally, research has demonstrated that autonomic dysfunction (AD) may cause aberrant esophageal and stomach motility, which may predispose one to develop GERD [28]. Figure 2 illustrates the link between obesity with both MASLD and GERD.

The association between GERD, MASLD, and AD

Autonomic dysfunction manifests as compromised sympathetic or parasympathetic symptoms. Prior studies have shown an association of AD with MASLD with increased mortality in those patients. However, there is insufficient data to establish a strong link between these two. Metabolic dysfunction-associated steatotic liver disease has a strong association with GERD, one of the many factors being the dysfunction of the autonomic nervous system. Furthermore, AD might predispose to abnormal esophageal and gastric motility, thus promoting the development of GERD and thereby increasing the risk of MASLD [29].

In a study of the middle-aged and elderly Chinese population, electrochemical skin conductance measurement (EZSCAN, Impeto Medical, Paris, FRA) was used to measure AD and correlate it with other risk factors. It is a non-invasive tool and highly specific to sweat gland function through sweat chloride measurement. In this large sample, it was found that patients with MASLD had a strong correlation, specifically a 38% higher risk of prevalent AD than those without MASLD. Age, BMI, systolic blood pressure (SBP), diastolic blood pressure (DBP), total cholesterol (TC), TG, high-density lipoprotein cholesterol (HDL-c), low-density lipoprotein cholesterol (LDL-c), current drinking, current smoking, liver enzymes, and prevalence of diabetes were significantly correlated with the AD index. These findings reinforced the importance of screening patients with MASLD for the presence of AD to facilitate timely intervention [30].

It has been proven that patients with MASLD have increased plasma FFA. In our body, reducing parasympathetic stimulation of white adipose tissue (WAT) increases the activity of hormone-sensitive lipase by almost 50%, which in turn increases the circulating FFA [31]. In the fed state, insulin suppresses the release of FFA. In a fasting state, glycogen reserves in the liver are broken down to generate adenosine triphosphate (ATP). This low liver glycogen level also stimulates increased hormone-sensitive lipase activity via sympathetic activation of lipolysis via hepatic vagal afferents. This pathway only applies to visceral fat, not the subcutaneous adipose tissue [32]. Therefore, both visceral and subcutaneous fat are controlled by different autonomic nerves arising from the hypothalamus and suprachiasmatic nucleus (SCN) [33]. Hence, AD or circadian dysfunction may predispose to raised FFA in MASLD [34].

Ultimately, the triaglycerides (TAG) released from the liver are transported via VLDL, which also has diurnal variations and is influenced by the timing of food intake. Increased parasympathetic stimulation causes a reduction in the release of VLDL, which contributes to the early stages of MASLD [34]. It is indicated that nearly 15% of TAG accumulation in the liver of MASLD patients originates directly from dietary sources and intake [35]. The enzyme lipoprotein lipase (LPL) is responsible for hydrolyzing the chylomicron containing TAG for metabolic use or deposition into the liver. The activity of LPL is high in a fed state, where insulin and parasympathetic-driven clearance of dietary-derived TAG is achieved. The LPL also decides how much FFA is stored in the liver and how much is released in the bloodstream. This, in turn, establishes a strong link with autonomic function. Other than this, for people with MASLD, nearly 25% of their liver TAGs arise from DNL [35]. This also has diurnal variations and has higher rates in the fed state. 

Studies have demonstrated that sympathetic stimulation of adipose tissue downregulates the production and secretion of adiponectin [36,37], while parasympathetic activation has the opposite effect. Therefore, in the context of MASLD, an imbalance with increased sympathetic and decreased parasympathetic input to adipose tissue could potentially lead to decreased adiponectin levels, contributing to the progression of the disease [34]. This emphasizes the intricate interplay between the autonomic nervous system and adiponectin regulation, providing insights into potential factors influencing NAFLD development and progression. Oxidative stress is also an important factor in disease progression. It increases sympathetic stimulation, which favors progression to NASH [34].

**Figure 3:**
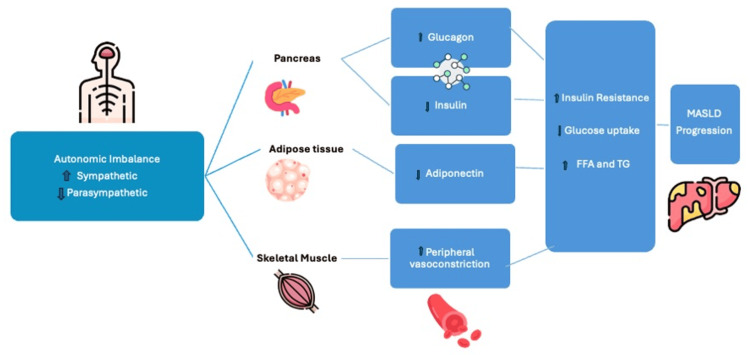
Autonomic dysfunction and its link with MASLD As shown by the illustration, the imbalance between both sympathetic and parasympathetic nervous systems alters the dynamics that lead to further progression of MASLD. MASLD: Metabolic dysfunction-associated steatotic liver disease, FFA: Free fatty acid, TG: Triglyceride Illustration created by the authors.

Insulin resistance mediated by the sympathetic nervous system (SNS), under the influence of hypothalamic neuropeptide Y and other contributing factors, leads to compensatory hyperinsulinemia and hyperglycemia in metabolic disease conditions. Consequently, this cascade contributes to the advancement of MASLD [38-40]. Furthermore, evidence indicates that SNS activity may directly impact HSCs, playing a role in the pathogenesis of MASLD [5]. Figure 3 shows the dynamic relationship between MASLD and AD.

Management

For patients with MASLD, a general risk assessment is done based on existing comorbidities. Primary risk is determined by the fibrosis-4 (FIB-4 index) and factors such as age, alanine transaminase (ALT), aspartate aminotransferase (AST), and platelet count. The NAFLD activity score (NAS) rates the components, i.e., steatosis (0-3), inflammation (0-3), and ballooning (0-2) with a total range of 0-8 and an improvement marker of ≥2 point reduction (≥1 in ballooning) without fibrosis progression. The imaging techniques used to study drug efficacy are vibration-controlled transient elastography (VCTE) using FibroScan (Echosens, Paris, FRA), magnetic resonance elastography (MRE), magnetic resonance spectroscopy (MRS), and MRI-derived proton density fat fraction (MRI-PDFF).

Elafibranor, a PPARα/δ agonist, showed promise in animal models and early human trials of the phase 2B GOLDEN study for improving MASLD symptoms. However, the phase 3 RESOLVE-IT study failed to demonstrate significant MASH resolution compared to placebo. While it improved some lipid profiles, elafibranor is no longer being pursued as a MASH treatment. Obeticholic acid (OCA), a potent synthetic FXR ligand, showed promise in the FLINT trial for MASH treatment. The OCA's mechanism involves FXR activation, which can reduce hepatic gluconeogenesis, lipogenesis, and steatosis. It improved necro-inflammation without worsening fibrosis in 45% of patients versus 21% in the placebo group, which is analogous to NAS>2. However, MASH resolution rates were not significantly different.

The OCA's main drawback is its negative impact on lipid profiles, increasing LDL-C and decreasing HDL-C. These effects normalized after discontinuation and were mitigated by atorvastatin in the CONTROL trial. Despite concerns, no short-term cardiovascular events were observed. Investigation of this drug has stopped. The HTD1801 (berberine ursodeoxycholate/BUDCA), which separates in the gut after ingestion, showed promise in a phase 2 trial for diabetic MASH patients. High-dose treatment led to greater liver fat reduction, LDL-C decrease, and weight loss compared to placebo. 

Tropifexor, a non-bile acid FXR agonist, was studied in the FLIGHT-FXR phase 2 trial for MASH treatment. It reduced AST, ALT, HDL-C, and liver fat but increased LDL-C. Higher doses (140µg and 200µg) achieved greater hepatic fat reduction than placebo. However, MASH resolution and NAS improvement rates were low across all groups. Dose-dependent pruritus was the main side effect. Despite the initial promise of fat reduction, the study was ultimately terminated due to insufficient efficacy in resolving MASH. Cenicriviroc (CVC), a dual CCR2/CCR5 antagonist, was investigated for MASLD treatment due to elevated CCL2 levels in MASH patients. The CENTAUR phase 2b study showed initial promise, but extended treatment did not improve fibrosis compared to placebo. The subsequent AURORA phase 3 trial found no fibrosis improvement after 12 months of CVC 150 mg daily despite good tolerability. Consequently, the AURORA trial was terminated [41].

The MAESTRO-NASH phase 3 trial resmetirom, an oral selective thyroid hormone receptor beta agonist, demonstrated both fibrosis improvement and MASH resolution. This success led to its FDA approval in March 2024, making it the first approved therapy for non-cirrhotic MASH patients with moderate to advanced (F2-F3) fibrosis. Resmetirom showed efficacy in biopsy-proven MASH with liver fibrosis. Both 80 mg and 100 mg doses outperformed placebo in improving MASLD activity score and fibrosis stage. Benefits extended across subgroups and included improvements in lipid profiles, liver biochemistry, and non-invasive assessments. The drug was generally safe, with mild gastrointestinal side effects at the start of treatment. Serious adverse event rates were similar to placebo, ranging from 10.9% to 12.7% across all groups [42].

Other therapeutic options include drugs that address the cardiometabolic risk factors. While metformin has been studied, the results, however, are inconclusive in terms of MASLD resolution. Liraglutide was studied in the phase 2 LEAN study, which showed less progression of fibrosis without any significant change in the TG, TC, HDL-C, and LDL-C at the end of 48 weeks. The SGLT inhibitors such as empagliflozin studied in the E-LIFT trial demonstrated a reduction in liver fat, although with a concurrent increase in LDL level. Ezetimibe in the MOZART study was unsuccessful in improving the NAS score or hepatic steatosis significantly. Pioglitazone, on the other hand, has shown improvement in liver enzymes and hepatic insulin sensitivity with decreased hepatic fat reduction. However, fibrosis was not significantly improved. It has been proposed by the American Association for the Study of Liver Diseases (AASLD) guidelines as a treatment for biopsy-proven MASH who also have diabetes with weight gain as a side effect. Moreover, MASLD treatment prioritizes lifestyle changes, including weight reduction, increased physical activity, and improved diet.

## Conclusions

Metabolic dysfunction-associated steatotic liver disease, previously known as NAFLD, is the most prevalent hepatic manifestation of metabolic syndrome. In recent years, it has shown a strong association with GERD. The link between these conditions is best explained by alterations in gastroesophageal physiology.

Several risk factors contribute to this association, including obesity, gender, lifestyle factors, AD, race, and Helicobacter pylori infection. Visceral obesity, especially, has emerged as a crucial risk factor strongly connected to both MASLD and GERD. The progression of MASLD tends to be more pronounced in obese patients, particularly in males. Elevated levels of inflammatory cytokines such as IL-1, IL-6, TNF-alpha, and TGF-beta, along with sympathetic stimulation, directly contribute to disease progression. Insulin resistance, which is positively mediated by sympathetic overdrive, further corresponds to the progression of MASLD. This proinflammatory state, coupled with sympathetic overdrive and obesity, also contributes to the symptoms of GERD. Consequently, obesity stands out as an independent risk factor for GERD as well as MASLD.
